# Effects of stretching in a pilates program on musculoskeletal fitness: a randomized clinical trial

**DOI:** 10.1186/s13102-024-00808-6

**Published:** 2024-01-08

**Authors:** Alex Lopes dos Reis, Laís Campos de Oliveira, Raphael Gonçalves de Oliveira

**Affiliations:** 1 Postgraduate Program in Human Movement Sciences, Health Sciences Center, Universidade Estadual do Norte do Paraná (UENP), Alameda Padre Magno, 841, Nova Alcântara, Jacarezinho, PR CEP: 86400-000 Brazil; 2 Postgraduate Program in Physical Exercise in Health Promotion, Health Sciences Research Center, Universidade Norte do Paraná (UNOPAR), Londrina, PR Brazil

**Keywords:** Young adult, Warm-Up exercise, Physical fitness, Exercise movement techniques

## Abstract

**Background:**

The scientific literature questions the impact of stretching exercises performed immediately before muscle strengthening exercises on different components of musculoskeletal physical fitness. Pilates is a physical exercise modality that typically uses stretching exercises preceding muscle-strengthening exercises. However, no studies have investigated the effects of stretching in a Pilates program on components of musculoskeletal fitness. The aim of the present study was to verify the effects of stretching in a Pilates exercise program on flexibility, strength, vertical jump height and muscular endurance.

**Methods:**

Thirty-two sedentary young women were randomized into two groups: traditional Pilates (TP), who performed flexibility and muscle strengthening exercises (*n* = 16), and nontraditional Pilates (NTP), who only performed muscle-strengthening exercises (*n* = 16). Sessions took place 3 times a week for 8 weeks. The following tests were performed pre- and postintervention: 10-RM knee extensors, vertical jump, handgrip, 1-min sit-ups, Sorensen and sit-and-reach. The occurrence of adverse events was recorded throughout the intervention and compared between groups using odds ratio (OR). To compare the results of motor tests between groups, ANCOVA or Mann‒Whitney U test was used for parametric and nonparametric data, respectively. The data were analyzed by intention-to-treat.

**Results:**

After intervention, the TP was superior to NTP for the sit-and-reach test, with a large effect size (d = 0.87; *p* = 0.035), with no differences between groups for the other tests. Intragroup comparisons showed significant differences (*p* < 0.05) for TP and NTP for improvement in 10-RM knee extensors and vertical jump measurements, while only TP showed significant intragroup improvement (*p* < 0.05) for the sit-and-reach test. A greater chance of experiencing pain or other discomfort as a result of exercise was shown by NTP (OR = 4.20, CI_95%_ 0.69 to 25.26).

**Conclusion:**

Our findings demonstrated that stretching exercises performed at the beginning of sessions in a Pilates program did not impair or enhance the development of strength, vertical jump height and muscular endurance in young women. However, only the Pilates program with stretching improved flexibility and reduced the chances of adverse events such as musculoskeletal pain and other discomfort resulting from the exercise protocol.

**ClinicalTrials.gov:**

NCT05538520, prospectively registered on September 16, 2022.

**Supplementary Information:**

The online version contains supplementary material available at 10.1186/s13102-024-00808-6.

## Background

Stretching exercises are widely used in training routines, typically preceding or following main exercise practice, whether the objective is related to rehabilitation [1] or physical conditioning [2], also aiming at improving performance in subsequent exercises involving musculoskeletal fitness [3]. In addition, stretching is considered the main exercise to gain flexibility [4], although resistance training alone can promote increased flexibility [5]. However, the performance of stretching exercises, preceding muscle strengthening exercises, has been questioned in the literature over the last few years [6–8], which evidently can generate doubts in the definition of intervention protocols for the most diverse objectives.

For example, even considering that static stretching or techniques such as proprioceptive neuromuscular facilitation are effective for improving flexibility, studies show that performing these forms of stretching, preceding muscle strengthening training, can generate a significantly lower gain in strength, power and muscular resistance when compared to its nonperformance [9–11]. On the other hand, the literature has noted that dynamic stretching does not seem to impair the subsequent development of these components of musculoskeletal fitness and may, in some cases, enhance its gain [12, 13]. However, most studies within this theme involve acute interventions, which may not reflect long-term effects [14]. Furthermore, some forms of stretching have not been investigated, such as the stretching exercises used in the practice of Pilates, which despite having a dynamic characteristic, are performed at a slow pace, following principles such as concentration, breathing, control, precision, centralization and fluidity [15].

It is common in Pilates protocols for stretching exercises to be performed at the beginning of the session, preceding muscle strengthening exercises [16–18]. However, how this impacts a possible harm or benefit in the development of variables such as strength, power and muscular endurance is unknown. Pilates is a technique used both in cases of rehabilitation and for physical conditioning, involving stretching exercises and muscle strengthening [15, 19]. The popularity of the technique has increased; however, scientific research is still in its infancy. In the USA, for example, between 2010 and 2021, an average of 10 million Pilates practitioners were registered per year [20]. In Brazil, when grouped alongside yoga, gymnastics and stretching, it is characterized as the sixth most practiced physical activity [21].

Although Pilates exercises are often associated with the treatment of low back pain and have been demonstrated to be effective for this purpose [22], in recent years, the technique has been explored for different applications, such as observing its effectiveness in the musculoskeletal fitness of volleyball [23] and basketball [24] players. Understanding the impact of stretching exercises used in Pilates on components of musculoskeletal fitness can help physical exercise professionals define intervention protocols, whether aimed at rehabilitation or physical conditioning. With this issue in mind, the aim of the present study was to verify the effects of stretching in a Pilates program on flexibility, muscle strength, vertical jump height and endurance.

## Methods

### Study design

This is a randomized clinical trial (prospectively registered in ClinicalTrials.gov ID NCT05538520) that followed the recommendations of CONSORT [25]. The randomization process was performed by a blinded researcher who was not part of the study team. Random numbers were generated by online software (randomization.com), which distributed participants into two groups of equal size (1:1). The same researcher who performed the randomization process sealed the opaque envelopes containing the group that each participant would be allocated and handed it to the main researcher. Subsequently, blind participants individually received the envelope numbered according to their entry into the study and broke the seal to verify which would be their allocation group. In this study, because it is an intervention with exercise, it was not possible to blind the participants and the therapist responsible for the intervention.

### Sample size

The sample calculation was performed using the Bioestat 5.3 program (Instituto Mamirauá, Amazonas, Brazil), taking into account the muscle strength values of the lower limbs available in a previous study [26]. In this case, the postintervention mean and standard deviation for muscle strength of the lower limb evaluated by the 10-RM test (kg) in the Leg Press between the group “dynamic stretching combined with resistance training” (337.1 ± 37.29) vs. the group “resistance training alone” (374.3 ± 32.07) was used, with test power at 80% and alpha value at 0.05, which generated the need for at least 16 participants in each group, already considering an additional 15%, to avoid a decrease in statistical power in case of sample loss.

### Participants

The sample consisted of 32 female participants who were insufficiently active and apparently healthy; the participants were subdivided into two groups of equal size (Table 1). Recruitment was carried out in the city of Ibaiti, state of Paraná, Brazil, through poster advertisements posted in public places and advertisements on social media. The ethical norms established in the Declaration of Helsinki (1975, revised in 1983) were followed, and the study was approved by the Human Research Ethics Committee of the Universidade Estadual do Norte do Paraná, Brazil, before its beginning under the opinion number 5.548.126. All participants signed an informed consent form.

**Table 1:** Baseline characteristics of participants

	TP (*n* = 16)	NTP (*n* = 16)
Age (years)	27.44 (5.70)	29.00 (7.59)
Body mass (kg)	56.06 (5.99)	55.47 (4.45)
Height (cm)	160.69 (5.95)	161.69 (4.79)
BMI (kg/m^2^)	21.64 (1.49)	21.19 (1.76)

Data are expressed as the mean and standard deviation

TP: Traditional Pilates; NTP: Non-Traditional Pilates; BMI: body mass index

Inclusion criteria were as follows: a) being aged between 18 and 45 years old; (b) having a body mass index (BMI) between 18.5 and 24.9 kg/m^2^ (normoweight individuals); (c) not participating in physical exercise programs for at least six months; (d) being healthy, according to the Physical Activity Readiness Questionnaire (PAR-Q) [27]; (e) not reporting any known medical restrictions on physical exercise; (f) having no history of injury, trauma or illness in the last six months; (g) not having undergone previous surgery in the last six months; (h) not having cardiorespiratory and neurological musculoskeletal disorders; (i) not being under the action of drugs that cause muscle relaxation or that may inhibit muscle tonic action; (j) not using dietary supplements or anabolic steroids; (k) not being on a calorie-restricted diet; and (l) being insufficiently physically active according to the IPAC short version [28].

Exclusion criteria were: (a) starting the practice of any type of physical exercise during the study period; (b) refusal to participate in assessment or intervention procedures; (c) emergence of injuries or other intercurrence during the intervention period; (d) withdrawal from participating in the study.

### Assessment procedures

Motor tests chosen as indicators of strength, vertical jump height, muscular endurance and flexibility were administered. These tests were administered at two times: (1) in the week before the interventions (preintervention assessment) and (2) in the week following the end of the interventions (postintervention assessment). Prior to the motor tests, all participants underwent a 10-minute warm-up on a cycle ergometer (60 rpm, 80 W). The same evaluator performed all motor tests, respecting a five-minute interval between each test, and the tests were performed in the same order of execution for all participants pre- and postintervention.

#### 10-RM knee extensors

The muscle strength of the knee extensors was assessed using elastic bands (Thera Band GmbH, Hadamar, Germany) as previously described [29]. Three elastic bands (black, silver and gold) were used, which, depending on the number of layers and initial length, allowed fifteen different resistances, represented in kg, known from the percentage of displacement (Table 2) [29, 30]. The participants were positioned on the seat of a standardized chair and were tested on their dominant side. For the evaluation, the elastic band was attached immediately proximal to the lateral malleolus of the participants and on a backrest positioned behind the chair (~ 40 cm below the knee and ~ 160 cm behind this articulation). Participants were asked to perform the maximum number of repetitions with an elastic band of intermediate resistance (black). If they were able to perform 11 repetitions, an elastic band of greater resistance was selected for the next attempt. This procedure was repeated (maximum of 5 attempts with 5 min of rest between attempts) until the participant was able to perform 10 or fewer repetitions. The following prediction equation was then used: 1-RM = resistance in kg/(1.0278 – [0.0278 × reps]) [31]. Therefore, a 10-RM test was performed dynamically and with consecutive repetitions, but the values were subsequently adjusted to represent the 1-RM according to the procedure indicated in the validation study of this test [29]. This test demonstrated high validity against peak concentric exchange measured by an isokinetic dynamometer (*r* = 0.93) and test-retest reliability (ICC ≥ 0.98, CV [%] ≤ 3.44) [29].

**Table 2:** Resistance of elastic bands for assessments of the 10-RM knee extensors

Thera-Band color	Number of layers	Initial length (cm)	Knee Extensors	
			Terminal length (%)	Resistance (kg)
Black	4	70	160	22.9
		65	180	25.0
		60	203	27.5
Silver	4	75	143	29.5
		70	160	32.2
		65	180	35.3
		60	203	38.9
		55	231	43.2
Gold	4	75	143	47.4
		70	160	51.7
		65	180	56.5
		60	203	62.3
		55	231	69.0
		50	264	77.1
		45	304	87.0

Adapted from Guex, Daucourt and Borloz [29]

#### Vertical jump

The countermovement jump test (sargent jump) was used to measure vertical jump height in cm. The objective of the test was to verify the highest height at the peak of the vertical jump, measuring two points marked on the wall by the participant himself, with the fingers of one of the hands dirty with chalk: (1) the initial measurement was obtained with the participant standing and erect, keeping the dominant arm extended parallel and above the head, marking the wall with chalk as high as possible, keeping the soles of the feet fully in contact with the floor; (2) the second measurement was obtained with the participant touching the wall with the fingers of the hand as high as possible during the peak of the vertical jump. Three attempts were allowed with intervals of two minutes between them, and the highest value in centimeters among the three attempts was recorded. This test demonstrated high validity (*r* = 0.99) and test-retest reliability (*r* = 0.99) [32].

#### Handgrip

To determine handgrip strength, a digital hydraulic dynamometer (Saehan SH1001) was used. The participant had to be in a sitting position, with an erect spine and knees flexed at 90°. The dominant upper limb tested was positioned with the shoulder in adduction and neutral rotation, wrist in neutral position and elbow at a 90° angle. Participants were instructed to perform three repetitions of maximum contraction maintained for five seconds, with a 60-second interval between each repetition. The maximum voluntary contraction was determined by the highest value obtained in kg from the three attempts. This instrument demonstrated excellent validity (*r* ≥ 0.97) and test-retest reliability (*r* ≥ 0.98) [33].

#### 1-min sit-ups

To evaluate abdominal muscle endurance, the 1-min sit-ups test was performed. In dorsal decubitus, the participant had to flex her knees, keeping the soles of her feet in contact with the ground, with her feet separated at a distance identical to the width of her hips. Her arms were crossed over her chest. The evaluator rested his hands on the participant’s feet to keep them in permanent contact with the ground. At the signal emitted by the evaluator, the participant raised the trunk until the anterior face of the forearms came into contact with the thighs, returning shortly afterwards to the initial position, with the contact of at least the anterior half of the scapulae on the ground. These movements had to be repeated over the course of one minute. The result was recorded as the number of correct repetitions performed in one minute. This test demonstrated high test-retest reliability (ICC = 0.91) [34].

#### Sorensen

To evaluate the muscular endurance of the trunk extensors, the Sorensen test was used. The participant had to be in ventral decubitus with the lower extremity of the body affixed to a stretcher, with the region of the anterior superior iliac crest demarcating the final point of support. Before starting the test, the participant was allowed to rest the upper extremity of the body, located outside the stretcher, on a chair. The test started when the participant raised her torso and remained without leaning on the chair, with her arms crossed in front of her chest, and kept her torso parallel to the ground for as long as possible. The test was interrupted when the participant could no longer sustain the position or was warned more than 2 times to align the trunk and maintain the neutral position. The test results were recorded within seconds. This test demonstrated moderate to high test-retest reliability in healthy adults (ICC ≥ 0.76 ≤ 0.97) [35].

#### Sit-and-reach

To assess the flexibility of the posterior region of the body, the sit-and-reach test with the Wells bench was used. The participant had to sit facing the bench, with bare feet, knees extended and the soles of the feet in contact with the bench. The participant extended her arms over the surface of the bench, with her hands positioned one over the other, coinciding with the tip of her middle fingers. Trunk forward flexion was requested, keeping the arms extended and the hand touching the measurement scale, trying to reach the greatest possible distance in a slow movement and without jerks. For the purpose of the final result of the test, the greatest distance in centimeters achieved in the series of three attempts was computed. This test demonstrated high test-retest reliability (*r* = 0.98) [36].

### Pilates protocol

The intervention consisted of 24 sessions of Pilates exercises performed three times a week for 8 weeks. Each session lasted 50 min in the Traditional Pilates (TP) group and 40 min in the Non-Traditional Pilates (NTP) group. The sessions took place on Mondays, Wednesdays and Fridays in the afternoon. The equipment used to perform the exercises was as follows: Step Chair, Cadillac Trapezio, Reformer Universal and Ladder Barrel (ISP, Brazil).

The TP and NTP protocols were identical, except for the fact that TP performed stretching exercises at the beginning of the sessions and NTP did not (Table 3). TP performed a total of 20 exercises, while NTP performed 15 exercises. Participants were instructed to follow the principles of Pilates (center, control, concentration, fluidity, precision and breathing). The Pilates protocol containing the images of the exercises is available as a supplementary file.

**Table 3:** Sequence of Pilates exercises

Type of exercise	Exercise name	Equipment/ accessory
Stretching*	Stretching the Chain Posterior	Reformer
	Front Splits Modified	Reformer
	Stomach Massage: Round	Reformer
	Stretches Front	Ladder Barrel
	Stretches Back: Quadriceps Stretch	Ladder Barrel
Core Strengthening	Bridge	Magic Circle/Ball
	The Hundred	Ball
	Teaser	Mini Barrel
	Swan	Mini Barrel
	Swimming	Mini Barrel
Lower limbs strengthening	Footwork Double Leg Pumps	Step Chair
	Pump One Leg Front	Step Chair
	Forward Lunge	Step Chair
	Wall Side	Ball
	Tower	Cadillac
Upper limbs strengthening	Arms Pulling I	Cadillac
	Rowing Front: Hug a Tree	Cadillac
	Arms Pulling II	Cadillac
	Arm Pulling III	Cadillac
	Extension Arm Up	Cadillac

*Stretching exercises are only performed at the beginning of the sessions by the Traditional Pilates group. The remaining exercises were performed by both groups (Traditional Pilates and Non-Traditional Pilates), always maintaining the same sequence

The exercises were performed in a series of ten repetitions, with a one-minute rest interval between exercises. For load progression, the resistance of the springs in each piece of equipment was replaced (changing the position of the springs in the equipment or replacing the springs with others of greater resistance) every 15 days, as the evolution of the strength of the participants was observed. The number of sets, repetitions and rest intervals were always maintained.

To determine the level of effort and consequently the evolution of loads, verbal descriptions were used according to the OMNI scale [37]: extremely easy (OMNI 0–1), easy (OMNI 2–3), reasonably easy (OMNI 4–5), fairly difficult (OMNI 6–7), difficult (OMNI 8–9) and extremely difficult (OMNI 10). The effort level maintained during the sessions was between 8 and 9 (difficult). In all sessions, the participants were asked about possible adverse events resulting from the interventions. Any reported adverse events were noted on the individual form used to record the training.

### Data analysis

Data normality was verified using the Shapiro‒Wilk test. To verify whether the groups presented differences at the beginning of the study, Student’s t test for independent samples was used. Intragroup alterations pre and postintervention were analyzed using Student’s t test for dependent samples for parametric data and the Wilcoxon test for nonparametric data. To verify differences between groups, analysis of covariance (ANCOVA) was performed, with postintervention data used as the dependent variable and preintervention data as the covariate. The homogeneity of variances was determined by Levene’s test. For nonparametric data, differences between groups at postintervention were calculated using the Mann‒Whitney U test.

Within-group and between-group effect sizes were calculated using Cohen’s d, which was considered trivial (0-0.19), small (0.20–0.49), medium (0.50–0.79) or large (≥ 0.80) [38]. Data were analyzed per protocol and by intention-to-treat analysis (ITT). As the results did not change, we present only the ITT data, including all randomized individuals (for missing postintervention data, preintervention data were imputed). To compare each adverse event reported between groups, the chi-square test was used. To determine the difference between groups in terms of the likelihood of developing any adverse events, odds ratio (OR) was calculated. For all tests, the significance level adopted was *P* < 0.05. Analyses were processed in SPSS 22.0 (Chicago, Illinois), except for effect size calculations (Cohen’s d), which were processed in GPower 3.1 (Franz Faul, Universität Kiel, Germany).

## Results

**Fig. 1:**
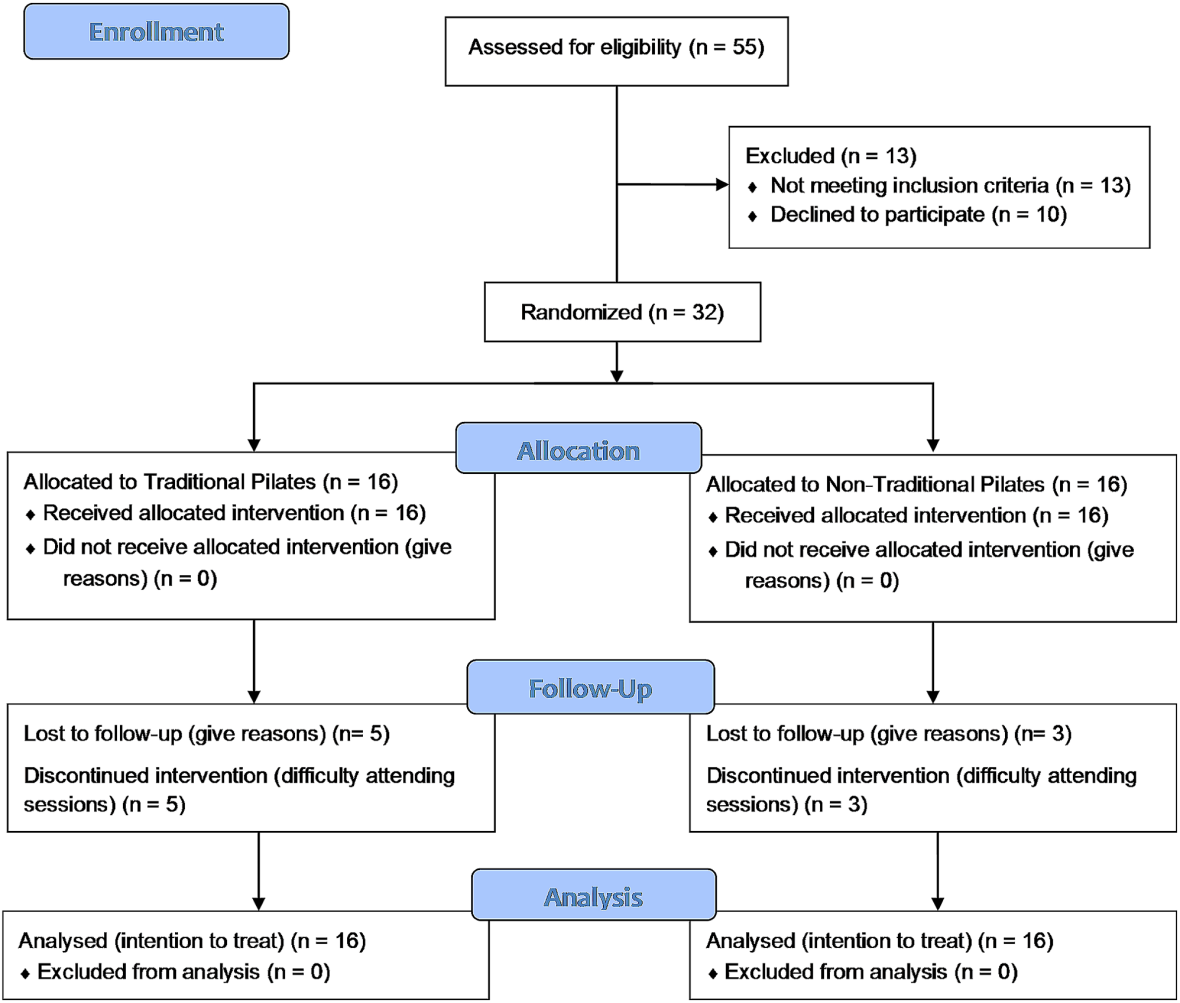
Flow diagram (CONSORT)

Initially, 55 participants were checked for eligibility (Fig. 1). Recruitment started on September 05, 2022, and the study was completed on November 25, 2022. Of the 32 participants who met the inclusion criteria and were randomized, there was a total loss of 8 participants throughout the study due to factors that made it unfeasible to continue the interventions, considering personal reasons and the emergence of commitments at the time of the interventions.

No significant (*p* > 0.05) difference between groups was observed at baseline for age, anthropometric variables of weight, height and BMI, and musculoskeletal fitness outcomes. Table 4 present the results of the intragroup and intergroup comparisons for the musculoskeletal fitness variables. In the intergroup comparison, the results showed a significant increase only for the sit-and-reach test, in favor of the PT (*p* = 0.03), with a large effect size (d = 0.87). In the intragroup comparison, significant results were observed in the following tests: 10-RM knee extensors in PT (*p* = 0.006, d = 0.79) and NTP (*p* = 0.01, d = 0.68); vertical jump in TP (*p* = 0.007, d = 0.78) and NTP (*p* = 0.001, d = 0.98); and sit-and-reach, only in PT (*p* = 0.003, d = 0.88). For handgrip, 1-min sit-ups and Sorensen tests, no significant results were found (*p* > 0.05).

**Table 4:** Intra- and intergroup comparisons for musculoskeletal fitness between TP vs. NTP

	TP (*n* = 16)	NTP (*n* = 16)	Cohen’s d	P‡
			(intergroup)	
10-RM knee extensors (kg)*				
Baseline	45.53 (14.66)	51.14 (10.77)	0.03	0.576
Post-intervention	52.94 (13.92)	58.18 (8.80)		
Change after 8 weeks	7.40 (9.35)	7.03 (10.31)		
Cohen’s d (intragroup)	0.79	0.68		
P (intragroup)	**0.006**	**0.01**		
Vertical jump (cm)				
Baseline	29.22 (3.40)	32.13 (5.51)	0.07	0.810
Post-intervention	31.34 (4.13)	34.44 (6.04)		
Change after 8 weeks	2.12 (2.72)	2.31 (2.35)		
Cohen’s d (intragroup)	0.78	0.98		
P (intragroup)	**0.007**	**0.001**		
Handgrip (kg)				
Baseline	28.15 (4.58)	30.03 (5.24)	0.32	0.238
Post-intervention	27.46 (5.12)	30.33 (5.01)		
Change after 8 weeks	-0.69 (2.58)	0.30 (3.47)		
Cohen’s d (intragroup)	0.27	0.09		
P (intragroup)	0.301	0.729		
1-min sit-ups (reps)				
Baseline	18.13 (7.35)	20.13 (7.14)	0.39	0.224
Post-intervention	19.56 (7.87)	20.56 (7.03)		
Change after 8 weeks	1.44 (3.28)	0.44 (1.50)		
Cohen’s d (intragroup)	0.42	0.20		
P (intragrupo)†	0.092	0.237		
Sorensen (sec)				
Baseline	61.38 (30.88)	63.75 (20.08)	0.22	0.390
Post-intervention	59.75 (29.74)	66.56 (14.22)		
Change after 8 weeks	−1.63 (21.64)	2.81 (17.94)		
Cohen’s d (intragroup)	0.07	0.16		
P (intragroup)	0.768	0.540		
Sit-and-reach (cm)				
Baseline	23.13 (6.43)	26.69 (8.95)	0.87	**0.035**
Post-intervention	27.31 (6.98)	27.50 (9.40)		
Change after 8 weeks	4.19 (4.76)	0.81 (2.78)		
Cohen’s d (intragroup)	0.88	0.29		
P (intragroup)	**0.003**	0.262		

10-RM (10 repetition maximum). *Values were converted to represent 1-RM (one repetition maximum) [29]. Data are expressed as the mean and standard deviation. TP: Traditional Pilates; NTP: Non-Traditional Pilates. Dependent Student’s t test for intragroup comparisons, except for 1-min sit-ups (†Wilcoxon test); ‡ANCOVA with preintervention data as a covariate, except for 1-min sit-ups (Mann‒Whitney U Test). Significant differences are highlighted in bold

### Adverse events

In total, 55 adverse events were reported—21 in the TP and 34 in the NTP—with the incidences being reported by 10 participants in the TP and 14 in the NTP. The chances of participants who did not stretch (NTP group) having an adverse event were four times greater (OR = 4.20, CI_95%_ 0.69 to 25.26). The participants’ main complaint was delayed onset muscle soreness, reported by PT (56.25%) and NTP (81.25%), mainly in the first weeks of intervention. Other adverse events, such as muscle pain in specific regions of the body (lumbar and cervical spine), dizziness, cramps, eagerness and nausea, occurred less frequently and were usually reported after exercise sessions (Table 5).

**Table 5:** Frequency of adverse events

	TP (*n* = 16)	NTP (*n* = 16)	P
Pain, n (%)			
Muscle soreness	9 (56.2)	13 (81.2)	0.127
Muscle pain in lower limbs	3 (18.7)	4 (25.0)	0.669
Muscle pain in the lumbar spine	0 (0.0)	1 (6.2)	0.310
Muscle pain in the cervical spine	1 (6.2)	0 (0.0)	0.310
Abdominal muscle pain	3 (18.7)	2 (12.5)	0.626
Muscle pain in upper limbs	2 (12.5)	4 (25.0)	0.365
Shoulder joint pain	2 (12.5)	3 (18.7)	0.626
**Others, n (%)**			
Dizziness	0 (0.0)	1 (6.2)	0.310
Cramp	1 (6.2)	1 (6.2)	1.000
Retching	0 (0.0)	3 (18.7)	0.069
Seasickness	0 (0.0)	2 (12.5)	0.144

Data expressed as absolute numbers (percentage values). The Chi-square test was used to compare groups. TP: Traditional Pilates; NTP: Non-Traditional Pilates

## Discussion

This study aimed to verify whether the practice of stretching in a Pilates program preceding strengthening exercises could affect the development of musculoskeletal fitness in sedentary young adult women. In brief, our findings demonstrated that only flexibility was affected. In this case, only the traditional Pilates program, which included stretching exercises at the beginning of the sessions, allowed an increase in the sit-and-reach test. For the indicators of strength, vertical jump height and muscular endurance, there was no difference between performing or not performing stretching exercises at the beginning of the sessions.

In this sense, the current study presents important practical applications, demonstrating that professionals who work with Pilates exercises can include stretching exercises at the beginning of sessions without compromising subsequent strengthening training, aiming to develop strength, vertical jump height and muscular endurance. Furthermore, if the objective is also to develop flexibility, professionals should include stretching exercises in the Pilates intervention protocol since performing strengthening exercises alone was not enough to improve flexibility. Our results also indicate that the five stretching exercises used in the protocol were exclusively responsible for improving flexibility in the TP group since only these exercises differed between the intervention groups.

This is the first long-term study that aimed to verify the effects of Pilates stretching exercises on musculoskeletal fitness components. Previous studies tested more traditional stretching, such as static, dynamic, ballistic and PNF, mainly in an acute condition, with few long-term studies [26, 39–42]. Notably, most long-term studies have focused only on intervening with stretching, aiming to verify its effects on various parameters, such as flexibility [40, 41], strength [40–42], vertical jump [39, 40] and muscular endurance [40]. Given that this is not the reality of most rehabilitation or physical conditioning programs, the present study used a protocol that reflects a common session in clinical practice in which stretching exercises and strengthening exercises are performed in the same session.

Previous studies that involved only stretching exercises have generally demonstrated the effectiveness of chronic stretching in increasing flexibility, muscular strength and vertical jump performance [39–42]. However, for muscular strength and vertical jump the effects are trivial to small, mainly benefiting sedentary people and older persons, while for flexibility, the effects are of moderate to large magnitude for any population [3]. In line with this perspective, in the present study, we observed that the five stretching exercises performed by the TP group had large effects (d > 0.80) on intra- and intergroup flexibility. However, we did not observe any effect of stretching on indicators of strength, muscular endurance and performance in the vertical jump.

A long-term study, which, like ours, sought to reproduce what is commonly done in clinical practice, that is, stretching and muscle strengthening exercises in the same session, found that after 12 weeks of intervention, there was no difference in flexibility and muscle strength of the upper limbs among the groups that performed (a) stretching before strength training; (b) stretching after strength training; (c) isolated strength training; and (d) isolated stretching. The only difference observed between groups was for lower limb muscle strength, in which the isolated strength training group was superior to the isolated stretching group [26]. That is, as in the present study, performing stretching and strengthening exercises in the same session did not affect muscle strength. However, as a divergence, in the current study, we found that only the group that performed Pilates stretching increased flexibility.

Unlike traditional dynamic stretching, in Pilates stretching exercises, the performer holds the final position of maximum joint amplitude for a few seconds, time necessary to inhale again, before returning to the initial position, performing exhalation. That is, the performer inhales whenever he is in the extremities (initial position and position of maximum amplitude) and exhales whenever he is in motion. This specific technique adopted by Pilates, in comparison with other forms of stretching, needs to be better explored in the literature. A study that compared Pilates exercises vs. static stretching identified after 12 weeks that the interventions did not differ for the following ranges of motion: trunk flexion, hip flexion, plantar flexion and dorsiflexion. However, Pilates exercises were superior for trunk range of motion in extension and for muscle strength of knee extensors and flexors [17, 18]. The present study advances the discussion by adding other indicators of musculoskeletal fitness in addition to flexibility, in addition to verifying the absence of stretching exercises in a Pilates program regarding these components.

Our findings help to fill an important gap in the scientific literature since little is known about the impact of performing Pilates stretching exercises preceding muscle strengthening exercises on flexibility and strength, vertical jump height and muscular endurance. The effects we found on flexibility in the group that performed Pilates stretching at the beginning of the sessions can be explained by the principle of specificity; that is, the exercises are directed toward this specific purpose. With this, an improvement in flexibility in the group that performed stretching was already expected, considering that this technique has already proven to be effective for this outcome in chronic interventions, if performed at least twice a week, regardless of the form of stretching [43].

Likewise, according to the principle of specificity, it was expected that after eight weeks of intervention, there would be a significant intragroup improvement, for both groups, in the results of the motor tests selected to evaluate muscular strength and endurance and performance in the vertical jump. This is because our protocol included 15 strengthening exercises for the main muscle groups. However, a significant improvement within groups occurred only for the 10-RM knee extensors and vertical jump tests, but not for the handgrip, 1-min sit-ups and Sorensen. To improve the muscle strength of the knee extensors and vertical jump performance, other clinical trials have also demonstrated the effectiveness of Pilates exercises [17, 44, 45].

In relation to the handgrip test, as it involves the isometric strength of a very specific region, such as the forearm and mainly palmar flexors, perhaps Pilates exercises, as they prioritize larger muscle groups, are not as effective at improving muscle strength in this region. This has also been observed in other clinical trials that did not find results for this outcome after the Pilates program [46, 47]. With regard to the muscular endurance of the trunk flexors and extensors, evaluated by the 1-min sit-ups and Sorensen tests, respectively, a probable justification for not observing significant results within the groups, be it the way the test is performed, compared with what is required in the practice of Pilates. In other words, while the Pilates exercise protocol requires muscular strength, the tests require muscular endurance.

Other studies that used these same tests also did not observe significant effects after a period of intervention with Pilates exercises [48, 49]. Unlike when measurement occurs through the muscular strength of the trunk flexor and extensor muscles through isokinetic dynamometry, for which Pilates has already proven effective [50]. Including enabling adequate balance of the muscles involved in flexion and extension of the trunk [51].

Finally, in relation to adverse events, although there was no significant difference between groups for each event individually, it was possible to observe that, in total, NTP presented 62% more adverse events when compared to TP. This represented a four times greater chance of developing an adverse event among participants who did not stretch. In this sense, the stretching exercises performed by the TP seem to have provided a protective effect on the sensation of pain and other discomfort resulting from the exercises. The lack of a significant difference between the groups for individual adverse events is probably related to the sample size, which was not scaled to meet this outcome. Although conflicting findings exist in the literature, a stretching routine immediately preceding physical exercise seems to contribute to the reduction of pain and discomfort [52].

The present study has some limitations that need to be highlighted: (a) we investigated only young women, as necessary studies also including male participants, as well as people of other age groups; (b) Pilates exercises were performed mainly on equipment, which may differ from a protocol exclusively designed with Mat Pilates exercises, in which the exercises are performed only with a gym mat, which may be investigated in future studies; (c) the vertical jump test and the 10-RM knee extensor and handgrip tests, from a biomechanical point of view, do not directly measure muscle power and strength, respectively; and (d) we did not explore the mechanisms that could explain the improvement in flexibility that occurred only in the TP group or the lack of difference between the groups for the other outcomes. Future studies should seek to replicate our study, if possible, while remedying the limitations presented.

## Conclusion

Our findings demonstrated that stretching exercises preceding strengthening exercises in a Pilates program did not impair, after 8 weeks of intervention, performance in tests indicative of strength, muscular endurance and vertical jump performance. Furthermore, the program that included stretching exercises was superior in improving flexibility and provided a lower chance of adverse events such as feelings of pain and other discomforts. In this sense, it is recommended that Pilates programs include stretching exercises prior to the muscle strengthening exercise routine, aiming to develop flexibility and reduce the sensation of pain and discomfort caused by the exercises.

### Electronic supplementary material

Below is the link to the electronic supplementary material.

**Supplementary Material 1**: Pilates Exercise Protocol

**Supplementary Material 2**: Study Protocol

## Data Availability

The datasets used and/or analysed during the current study are available from the corresponding author on reasonable request.
