# Supplementary material for: Effects of stretching in a pilates program on musculoskeletal fitness: a randomized clinical trial
Source: BMC Sports Sci Med Rehabil. 2024 Jan 8;16:11. doi: 10.1186/s13102-024-00808-6 (PMC10775508; doi:10.1186/s13102-024-00808-6)
Supplement: Supplementary file 1 — Supplementary Material 1: Pilates Exercise Protocol [file 13102_2024_808_MOESM1_ESM.docx]

**Supplementary File: Pilates Exercise Protocol**

**Stretching**

1- Stretching the Chain Posterior (Reformer).

2- Front Splits Modified (Reformer).

3- Stomach Massage: Round (Reformer).

4- Stretches Front (Barrel).

5- Stretches Back: Quadríceps Stretch (Barrel).

**Core Strengthening**

6- Bridge (Magic Circle): “a” initial exercises / “b” progression exercises.

7- The Hundred I (ball): “a” initial exercises / “b” progression exercises.

8- Teaser II (Mini barrel): “a” initial exercises / “b” progression exercises.

9- Swan IV (Mini barrel).

10- Swimming (Mini barrel).

**Lower limb strengthening**

11- Footwork Double Leg Pumps (Step Chair).

12- Pump One Leg Front (Step Chair).

13- Forward Lunge (Step Chair).

14- Wall Side (ball).

15- Tower (Cadillac).

**Strengthening of upper limbs**

16- Arms Pulling II (Cadillac).

17- Rowing Front: Hug a Tree I (Cadillac).

18- Arm Pulling III (Cadillac).

19- Arm Pulling V (Cadillac).

20- Extension Arm Up (Cadillac).
