# Supplementary material for: Effects of stretching in a pilates program on musculoskeletal fitness: a randomized clinical trial
Source: BMC Sports Sci Med Rehabil. 2024 Jan 8;16:11. doi: 10.1186/s13102-024-00808-6 (PMC10775508; doi:10.1186/s13102-024-00808-6)
Supplement: Supplementary file 2 — Supplementary Material 2: Study Protocol [file 13102_2024_808_MOESM2_ESM.pdf]

STUDY PROTOCOL

ADMINISTRATIVE INFORMATION

1. Title

Effects of Stretching in a Pilates Program on Musculoskeletal Fitness: A Randomized Clinical Trial

2. Trial registration

2a. ID ClinicalTrials.govNCT05538520

2b. Administrative Information

Other Study ID Numbers	Pilates_stretching
Has Data Monitoring Committee	No
U.S. FDA-regulated Product	Studies a U.S. FDA-regulated Drug Product: No<br>Studies a U.S. FDA-regulated Device Product: No
IPD Sharing Statement	Plan to Share IPD: No<br>Plan Description: Data regarding the study will be shared in the publication linked to this record, as a main part of the manuscript and supplementary documents when necessary.
Current Responsible Party	Raphael Goncalves de Oliveira, Universidade Estadual do Norte do Parana
Current Study Sponsor	Universidade Estadual do Norte do Parana
PRS Account	Universidade Estadual do Norte do Parana

3. Protocol version

1st version on 8/3/2023

4. Funding

This study was financed in part by the Coordenação de Aperfeiçoamento de Pessoal de Nível Superior – Brazil (CAPES) – Finance Code 001.

5. Roles and responsibilities

5a. Raphael Gonçalves de Oliveira (professor at the Universidade Estadual do Norte do Paraná, Brazil) and Alex Lopes dos Reis (Master's Scholarship by the Coordenação de Aperfeiçoamento de Pessoal de Nível Superior – Brazil).

5b. The study was partially sponsored by the Coordenação de Aperfeiçoamento de Pessoal de Nível Superior – Brazil. Address: Setor Bancário Norte (SBN), Block 2, Lot 06, CAPES Building – CEP: 70.040-020 – Brasília, DF, Brazil. E-mail: faleconosco@capes.gov.br

5c. The study sponsor has no influence on the study design; data collection, management, analysis and interpretation; report writing; and the decision to submit the report for publication.

5d. not applicable (Composition, roles, and responsibilities of the coordinating centre, steering committee, endpoint adjudication committee, data management team, and other individuals or groups overseeing the trial).

INTRODUCTION

6a. Background and rationale

Stretching exercises are widely used in training routines, typically preceding or following main exercise practice, whether the objective is related to rehabilitation [1] or physical conditioning [2], also aiming at improving performance in subsequent exercises involving musculoskeletal fitness [3]. In addition, stretching is considered the main exercise to gain flexibility [4], although resistance training alone can promote increased flexibility [5]. However, the performance of stretching exercises, preceding muscle strengthening exercises, has been questioned in the literature over the last few years [6–8], which evidently can generate doubts in the definition of intervention protocols for the most diverse objectives.

For example, even considering that static stretching or techniques such as proprioceptive neuromuscular facilitation are effective for improving flexibility, studies show that performing these forms of stretching, preceding muscle strengthening training, can generate a significantly lower gain in strength, power and muscular resistance when compared to its nonperformance [9–11]. On the other hand, the literature has noted that dynamic stretching does not seem to impair the subsequent development of these components of musculoskeletal fitness and may, in some cases, enhance its gain [12, 13]. However, most studies within this theme involve acute interventions, which may not reflect long-term effects [14]. Furthermore, some forms of stretching have not been investigated, such as the stretching exercises used in the practice of Pilates, which despite having a dynamic characteristic, are performed at a slow pace, following principles such as concentration, breathing, control, precision, centralization and fluidity [15].

It is common in Pilates protocols for stretching exercises to be performed at the beginning of the session, preceding muscle strengthening exercises [16–18]. However, how this impacts a possible harm or benefit in the development of variables such as strength, power and muscular endurance is unknown. Pilates is a technique used both in cases of rehabilitation and for physical conditioning, involving stretching exercises and muscle strengthening [15, 19]. The popularity of the technique has increased; however, scientific research is still in its infancy. In the USA, for example, between 2010 and 2021, an

average of 10 million Pilates practitioners were registered per year [20]. In Brazil, when grouped alongside yoga, gymnastics and stretching, it is characterized as the sixth most practiced physical activity [21].

Understanding the impact of stretching exercises used in Pilates on components of musculoskeletal fitness can help physical exercise professionals define intervention protocols, whether aimed at rehabilitation or physical conditioning.

6b. In order to be able to detect the influence of Pilates exercise stretching on musculoskeletal fitness components, it is necessary to adopt as a comparator a group that performs the same activities as the experimental group, however, without the addition of stretching exercises.

7. Objectives

To verify the effects of Pilates stretching exercises performed at the beginning of sessions on flexibility, strength, power and muscular endurance.

8. Trial design

Randomized and controlled clinical trial.

METHODS: PARTICIPANTS, INTERVENTIONS, AND OUTCOMES

9. Study setting:

Center for Health Sciences at the Universidade Estadual do Norte do Paraná, Brazil.

10. Eligibility criteria:

Inclusion Criteria

- ages between 18 and 45 years
- normoweights (body mass index between 18.5 to 24.9 kg/m^2)
- not be participating in physical exercise programs for at least six months
- be healthy, according to the Physical Activity Readiness Questionnaire
- not report any medical restrictions for physical exercise
- no history of injury, trauma or illness within the last six months
- not having undergone previous surgery in the last six months
- not have musculoskeletal, cardiorespiratory and neurological disorders that prevent the performance of assessment and intervention protocols
- not be under the action of medications that cause muscle relaxation or that can inhibit muscle tonic action
- not use food supplements or anabolic steroids

- not being on a calorie-restricted diet

Exclusion Criteria

- start practicing another type of physical exercise during the study period
- not being able to perform the pre-intervention assessment or the intervention exercise protocol
- emergence of lesions that do not allow the continuity of interventions
- withdraw from participating in the study

11. Interventions

11a. Intervention 1 – Traditional Pilates: This group will perform a traditional Pilates session, which consists of stretching exercises, followed by exercises to strengthen the core (trunk flexors and extensors), lower limbs and upper limbs. The total duration of the intervention in this group should be approximately 50 minutes.

Intervention 2 – Pilates without stretching: This group will conduct a non-traditional Pilates session. This is because stretching exercises will be excluded. The muscle strengthening exercises will be the same as in the TP group, so that it is possible to discuss the influence of the inclusion/exclusion of stretching for the considered outcomes. The total duration of the intervention in this group should be approximately 35 minutes.

11b. a participant must discontinue the intervention when at his own request (personal reasons), or due to any health problem that makes its continuity unfeasible (eg appearance of injury).

11c. adherence monitoring will occur through an attendance/absence form.

11d. all participants will be instructed to maintain their usual routine, especially with regard to physical activity and eating habits.

12. Outcomes

- Change in muscle strength of knee extensors and flexors [Time Frame: Pre (baseline) and post-intervention assessment (after 8-weeks)]. Assessment of muscle strength with elastic resistance
- Change in strength/endurance by 1-minute sit-up test [Time Frame: Pre (baseline) and post-intervention assessment (after 8-weeks)]. Assessment of abdominal muscle strength/endurance in 1-minute
- Change in muscle strength by handgrip test using hand dynamometer [Time Frame: Pre (baseline) and post-intervention assessment (after 8-weeks)]. Assessment of handgrip muscle strength
- Change in the muscular resistance of the trunk extensors by the Sorensen test [Time Frame: Pre (baseline) and post-intervention assessment (after 8-weeks)]. Assessment of the muscular resistance of the trunk extensors (Sorensen)

- Lower limb muscle power [Time Frame: Pre (baseline) and post-intervention assessment (after 8-weeks)]. Assessment of lower limb muscle power by vertical jump
- Flexibility [Time Frame: Pre (baseline) and post-intervention assessment (after 8-weeks)]. Flexibility assessment by the sit-and-reach test
- Affective valence [Time Frame: The scale will be applied at the end of each exercise session, over the 8 weeks, 3x a week, thus totaling 24 assessments throughout the study.]. Assessment of affective valence by the Feeling Scale. It ranges from +5 (very good) to -5 (very bad). Positive values represent pleasure, while negative values represent displeasure.

13. Participant timeline

Registration will remain open until the sample size is reached. Next, a week will be reserved for the initial assessments (pre-intervention), followed by eight weeks of intervention. Finally, in the following week, the final evaluations (post-intervention) will be carried out.

14. Sample size

The sample calculation was performed using the Bioestat 5.3 program (Instituto Mamirauá, Amazonas, Brazil), taking into account the muscle strength values of the lower limbs available in a previous study [22]. In this case, the postintervention mean and standard deviation for muscle strength of the lower limb evaluated by the 10-RM test (kg) in the Leg Press between the group "dynamic stretching combined with resistance training" (337.1 ± 37.29) vs the group "resistance training alone" (374.3 ± 32.07) was used, with test power at 80% and alpha value at 0.05, which generated the need for at least 16 participants in each group, already considering an additional 15%, to avoid a decrease in statistical power in case of sample loss.

15. Recruitment

Recruitment should occur mainly through disclosure on social media (internet).

METHODS: ASSIGNMENT OF INTERVENTIONS (FOR CONTROLLED TRIALS)

16. Allocation

16a. Sequence generation: The randomization process will be carried out by a blinded researcher who is not part of the study team. Random numbers will be generated by online software (randomization.com), which will distribute participants into two groups of equal size (1:1).

16b. Allocation concealment mechanism

The same researcher who performed the randomization process will seal the opaque envelopes containing the group that each participant will be allocated and deliver to the main researcher.

16c. Implementation

The sequence of allocation and enrollment of participants will be done by an independent researcher and the assignment of participants to interventions will be done by the main researcher.

17. Blinding (masking)

17a. As this is a clinical trial involving physical exercise, it will not be possible to blind participants and therapists. Only the rater is expected to be blind.

17b. To test whether the evaluator was really blind, the main researcher should question the evaluator after the evaluations regarding the allocation of each participant.

METHODS: DATA COLLECTION, MANAGEMENT, AND ANALYSIS

18. Data collection methods

18a. Data collection will be carried out by an experienced evaluator, a professional with a degree in Physiotherapy and Physical Education. The tests performed and their measurement properties are described below:

Muscle strength of the knee extensors and flexors – It will be evaluated using elastic bands (Thera Band GmbH, Hadamar, Germany) as previously described [23]. Participants will be tested on their dominant side. After a 10-minute warm-up on a cycle ergometer (60 rpm, 80 W), participants will be positioned on the seat of a standardized chair. Participants will be asked to perform the maximum number of repetitions with an elastic band of intermediate resistance (black color). If they managed to perform 11 repetitions, an elastic band of greater resistance will be selected for the next attempt. This procedure will be repeated (maximum of 5 attempts with 5 minutes of rest between attempts) until the participant is able to perform 10 or fewer repetitions. The following prediction equation will be used: 1-RM = resistance in kg/(1.0278 – [0.0278 × reps]) [24]. This test demonstrated high validity against concentric peak torque measured by an isokinetic dynamometer ($r = 0.93$) and test-retest reliability (ICC ≥ 0.98, CV[%] ≤ 3.44) [23].

Vertical jump – The countermovement jump test (sargent jump) will be used as an indicator of lower limb muscle power. The objective of the test will be to verify the highest height at the peak of the vertical jump, measuring two points marked on the wall by the participant himself, with the fingers of one of the chalk-stained hands: (1) the initial measurement will be obtained with the participant standing and erect, keeping the dominant arm extended parallel and above the head, marking the wall with chalk as high

as possible, keeping the soles of the feet completely in contact with the ground; (2) the second measurement will be obtained with the participant touching the wall with the fingers of the hand as high as possible during the peak of the vertical jump. Three attempts will be allowed with a two-minute interval between them, with the highest value in centimeters being recorded among the three attempts. This test demonstrated high validity (r = 0.99) and test-retest reliability (r = 0.99) [25].

Handgrip strength – To determine handgrip strength, a digital hydraulic dynamometer (Saehan SH1001) will be used. The participant must be seated, with the spine erect and the knees flexed at 90°. The dominant upper limb tested will be positioned with the shoulder in adduction and neutral rotation, wrist in neutral position and elbow at a 90° angle. Participants will be instructed to perform three repetitions of maximum contraction maintained for five seconds, with a 60-second interval between each repetition. The maximum voluntary contraction will be determined by the highest value obtained in the three attempts. This instrument demonstrated excellent validity (r ≥ 0.97) and test-retest reliability (r ≥ 0.98) [26].

Abdominal muscle endurance – To assess abdominal muscle endurance, the 1-minute sit-up test will be performed. In dorsal decubitus, the participant should flex the knees, keeping the soles of the feet in contact with the ground, with the feet apart at a distance identical to the width of the hips. Her arms should be crossed over her chest. The evaluator will place his hands on the participant's feet to keep them in permanent contact with the ground. At the signal emitted by the evaluator, the participant will raise the torso until the anterior face of the forearms comes into contact with the thighs, immediately returning to the initial position, with contact of at least the anterior half of the scapulae on the ground. These movements should be repeated over one minute. The objective of the test will be to try to perform as many executions as possible in the stipulated time. This test demonstrated high test-retest reliability (ICC = 0.91) [27].

Muscular resistance of the trunk extensors – To assess the muscular resistance of the trunk extensors, the Sorensen test will be used. The participant must be in ventral decubitus with the lower extremity of the body attached to a stretcher, with the region of the anterior superior iliac crest demarcating the final point of support. Before starting the test, the participant will be allowed to support the upper extremity of the body, located outside the stretcher, on a chair. The test will start when the participant raises the trunk and remains without touching the chair, with arms crossed in front of the chest, and keeping the trunk parallel to the ground as long as possible. The test will be stopped when the participant is unable to sustain the position or is prompted more than 2 times to align the trunk and maintain the neutral position. This test demonstrated moderate to high test-retest reliability in healthy adults (ICC ≥ 0.76 ≤ 0.97) [28].

Flexibility – To assess the flexibility of the posterior region of the body, the sit-and-reach test with the Wells bench will be used. The participant must sit facing the bench, with bare feet, knees extended and soles of the feet in contact with the bench. The participant should extend her arms over the surface of the bench, with her hands positioned one over the other, coinciding with the tip of her middle fingers. Anterior trunk flexion will be requested, keeping the arms extended and the hand touching the measurement scale, trying to reach the greatest possible distance in a slow movement and without jerks. For

the purpose of the final result of the test, the greatest distance achieved in the series of three attempts will be computed. This test demonstrated high test-retest reliability (r = 0.98) [29].

Affective valence – Assessment of affective valence will be performed by the Feeling Scale, which ranges from +5 (very good) to -5 (very bad). Positive values represent pleasure, while negative values represent displeasure. The answers will always be collected at the end of the exercise sessions. The adapted version of this scale for the Brazilian population showed high reproducibility (ICC = 0.64) [30].

18b. All randomized participants will be evaluated post-intervention considering the group to which they were initially allocated. All initially randomized participants will be analyzed post-intervention. An intention-to-treat analysis is therefore planned.

19. Data management

After the evaluations, the data will be tabulated and treated statistically by a researcher experienced in data analysis. To avoid possible errors, a second researcher should check the tabulated data and replicate the analyses.

20. Statistical methods

20a. Aiming at greater robustness and precision in data analysis, analysis of co-variance (ANCOVA) is planned, with baseline data used as co-variables, so that, strictly speaking, the effective differences between the groups in the post-intervention period are compared.

20b. In order to demonstrate the effects of the intervention considering only the subjects who completed the protocol, an analysis per protocol is also foreseen, even if in a secondary way.

20c. For the main analyses, performed on an intention-to-treat basis, imputations are anticipated for participants who do not complete interventions. In this case, data from the initial assessment (pre-intervention) will be replicated in the final assessment (post-intervention).

METHODS: MONITORING

21. Data monitoring

21a. For the present study, a data monitoring committee is not foreseen.

21b. No interim analysis is foreseen.

22. Harms

In all sessions, participants will be asked about the possible occurrence of adverse events.

23. Auditing

No audit is planned for this study.

ETHICS AND DISSEMINATION

24. Research ethics approval

The ethical norms established in the Declaration of Helsinki (1975, revised in 1983) were followed, and the study was approved by the Human Research Ethics Committee of the Universidade Estadual do Norte do Paraná, Brazil, before its beginning under the opinion number 5.548.126.

25. Protocol amendments

Any need to change the study protocol should be done via clinicaltrials.gov, which maintains an online record of changes that can be accessed by anyone.

26. Consent or assent

26a. The principal investigator will obtain informed consent from each participant. For this, he should explain in clear language all aspects of the study, such as: evaluation measures, intervention procedures, risks and benefits. In addition, a Consent Term approved by the Ethics Committee that approved the study must be read by the participant, who must sign the document if he/she wishes to participate in the study.

26b. If it is necessary to use the image of a person demonstrating the exercises of the intervention protocol, an informed consent will also be required specifically for this purpose.

27. Confidentiality

No personal data of participants will be kept in the study's digital databases. From the randomization process, the participants will be numbered and for purposes of data tabulation and analysis only these numbers will be used.

28. Declaration of interests

The study researchers declare that there are no conflicts of interest to declare for this work.

29. Access to data

The final data of the study were under the custody of the main researcher, who can provide access upon a formal request.

30. Ancillary and post-trial care

According to the project approved by the Ethics Committee, if any participant suffers damage as a result of the study (eg injury), he will receive free treatment until he improves.

31. Dissemination policy

31a. The results of the study should be published in a scientific journal (preferably with open access), in order to give a wide coverage to the findings.

31b. Eligibility for authorship will be defined according to the recommendations of the International Committee of Medical Journal Editors (ICMJE).

31c. Data referring to the protocol can be accessed via clinicaltrials.gov, while the spreadsheet containing data from pre- and post-intervention evaluations, as well as the statistical code, can be requested directly from the main researcher.

APPENDICES

32. Appendix to this protocol we forward the consent form approved by the local Ethics Committee.

33. No collection, laboratory evaluation, and storage of biological specimens for genetic or molecular analysis is applicable to this study.

REFERENCES

1. Luan L, El-Ansary D, Adams R, Wu S, Han J. Knee osteoarthritis pain and stretching exercises: a systematic review and meta-analysis. Physiotherapy. 2022;114:16-29. doi:10.1016/j.physio.2021.10.001
2. Babault N, Rodot G, Champelovier M, Cometti C. A Survey on Stretching Practices in Women and Men from Various Sports or Physical Activity Programs. Int J Environ Res Public Health. 2021;18(8):3928. doi:10.3390/ijerph18083928
3. Arntz F, Markov A, Behm DG, Behrens M, Negra Y, Nakamura M, Moran J, Chaabene H. Chronic Effects of Static Stretching Exercises on Muscle Strength and Power in Healthy Individuals Across the Lifespan: A Systematic Review with Multi-level Meta-analysis. Sports Med. 2023;53(3):723-745. doi: 10.1007/s40279-022-01806-9
4. Bouguezzi R, Sammoud S, Markov A, Negra Y, Chaabene H. Why Flexibility Deserves to Be Further Considered as a Standard Component of Physical Fitness: A Narrative Review of Existing Insights from Static Stretching Study Interventions. Youth. 2023; 3(1):146-156. doi: 10.3390/youth3010010
5. Leite TB, Costa PB, Leite RD, Novaes JS, Fleck SJ, Simão R. Effects of Different Number of Sets of Resistance Training on Flexibility. Int J Exerc Sci. 2017;10(3):354-364.
6. Peck E, Chomko G, Gaz DV, Farrell AM. The effects of stretching on performance. Curr Sports Med Rep. 2014;13(3):179-185. doi: 10.1249/JSR.0000000000000052
7. Simic L, Sarabon N, Markovic G. Does pre-exercise static stretching inhibit maximal muscular performance? A meta-analytical review. Scand J Med Sci Sports. 2013;23(2):131-148. doi: 10.1111/j.1600-0838.2012.01444.x
8. Gil MH, Neiva HP, Sousa AC, Marques MC, Marinho DA. Current Approaches on Warming up for Sports Performance: A Critical Review. Strength Cond J. 2019;41(4):70-79. doi: 10.1519/SSC.0000000000000454
9. Gomes TM, Simão R, Marques MC, Costa PB, da Silva Novaes J. Acute effects of two different stretching methods on local muscular endurance performance. J Strength Cond Res. 2011;25(3):745-52. doi: 10.1519/JSC.0b013e3181cc236a
10. Carvalho FLP, Prati JELR, Carvalho MCGA, Dantas EHM. Acute effects of static stretching and proprioceptive neuromuscular facilitation on the performance of vertical jump in adolescent tennis players. Fit Perform J. 2009;8(4):264-268. doi: 10.3900/fpj.8.4.264.e

11. Silva GVLC, Silveira ALBezerra, Masi FD, Bentes CM, Sousa MSC, Novaes JS. Acute effect of different stretching methods on isometric muscle strength. Acta Sci Health Sci. 2014;36(1):51-57. doi: 10.4025/actascihealthsci.v36i1.15581
12. Behm DG, Blazevich AJ, Kay AD, McHugh M. Acute effects of muscle stretching on physical performance, range of motion, and injury incidence in healthy active individuals: a systematic review. Appl Physiol Nutr Metab. 2016;41(1):1-11. doi: 10.1139/apnm-2015-0235
13. Opplert J, Babault N. Acute Effects of Dynamic Stretching on Muscle Flexibility and Performance: An Analysis of the Current Literature. Sports Med. 2018 Feb;48(2):299-325. doi: 10.1007/s40279-017-0797-9
14. Stone M, Ramsey MW, Kinser AM, O'Bryant HS, Ayers C; Sands WA. Stretching: acute and chronic? The potential consequences. Strength Cond J. 2006;28(6):66-74.
15. Wells C, Kolt GS, Bialocerkowski A. Defining Pilates exercise: a systematic review. Complement Ther Med. 2012;20(4):253-262. doi: 10.1016/j.ctim.2012.02.005
16. de Oliveira RG, Anami GEU, Coelho EA, de Oliveira LC. Effects of Pilates exercise on bone mineral density in postmenopausal women: a systematic review and meta-analysis. J Geriatr Phys Ther. 2022;45(2):107-114. doi: 10.1519/JPT.0000000000000309
17. Oliveira LC, Oliveira RG, Pires-Oliveira DAA. Pilates increases the isokinetic muscular strength of the knee extensors and flexors in elderly women. J Bodyw Mov Ther. 2017;21(4):815-822. doi: 10.1016/j.jbmt.2017.01.006
18. Oliveira LC, Oliveira RG, Pires-Oliveira DA. Comparison between static stretching and the Pilates method on the flexibility of older women. J Bodyw Mov Ther. 2016;20(4):800-806. doi: 10.1016/j.jbmt.2016.01.008
19. Di Lorenzo CE. Pilates: what is it? Should it be used in rehabilitation? Sports Health. 2011;3(4):352-361. doi:10.1177/1941738111410285
20. Statista. Number of pilates participants in the United States from 2010 to 2021. Available online: https://www.statista.com/statistics/191616/participants-in-pilates-training-in-the-us-since-2006. (Accessed May 20, 2023).
21. Guimarães Lima M, Malta DC, Monteiro CN, da Silva Sousa NF, Stopa SR, Medina LPB, de Azevedo Barros MB. Leisure-time physical activity and sports in the Brazilian population: A social disparity analysis. PLoS One. 2019;14(12):e0225940. doi: 10.1371/journal.pone.0225940

22. Leite T, de Souza Teixeira A, Saavedra F, Leite RD, Rhea MR, Simão R. Influence of strength and flexibility training, combined or isolated, on strength and flexibility gains. J Strength Cond Res. 2015;29(4):1083-1088. doi: 10.1519/JSC.0000000000000719
23. Validity and reliability of maximal-strength assessment of knee flexors and extensors using elastic bands. J Sport Rehabil. 2015;24(2):151-155. doi: 10.1123/jsr.2013-0131
24. Brzycki M. Strength testing: predicting a one-rep max from repetitions to fatigue. J Phys Educ Recr Dance. 1993;64:88-90. doi:10.1080/07303084.1993.10606684
25. de Salles PG, Vasconcellos FV, de Salles GF, Fonseca RT, Dantas EH. Validity and reproducibility of the sargent jump test in the assessment of explosive strength in soccer players. J Hum Kinet. 2012;33:115-21. doi: 10.2478/v10078-012-0050-4
26. Reis MM, Arantes PMM. Assessment of hand grip strength: validity and reliability of the saehan dynamometer. Fisioter Pesqui. 2011;18(2):176-181. doi: 10.1590/S1809-29502011000200013
27. DiNucci J, McCune D, Shows D. Reliability of a modification of the health-related physical fitness test for use with physical education majors. Res Q Exerc Sport. 1990;61(1):20-25. doi: 10.1080/02701367.1990.10607474
28. Keller A, Hellesnes J, Brox JI. Reliability of the isokinetic trunk extensor test, Biering-Sørensen test, and Astrand bicycle test: assessment of intraclass correlation coefficient and critical difference in patients with chronic low back pain and healthy individuals. Spine. 2001;26(7):771-777. doi: 10.1097/00007632-200104010-00017
29. Wells KF, Dillon EK. The Sit and Reach—A Test of Back and Leg Flexibility. Res Q Am Assoc Health Phys Educ. 1952;23(1):115-118. doi: 10.1080/10671188.1952.10761965
30. Alves ED, Panissa VLG, Barros BJ, Franchini E, Takito MY. Translation, adaptation, and reproducibility of the Physical Activity Enjoyment Scale (PACES) and Feeling Scale to Brazilian Portuguese. Sport Sci Health. 2019;15:329–336. doi: 10.1007/s11332-018-0516-4

APPENDIX. Term of Free and Informed Consent

Research title: Effects of stretching in a fitness program based on Pilates exercises on flexibility, strength, endurance and muscle power: a randomized clinical trial

You are being invited to participate, as a participant, in a survey. Please read what follows carefully and ask me any questions you may have. After being clarified about the following information, if you agree to take part in this study, sign at the end of this document and initial the first page. The document will be signed in duplicate. One copy belongs to you and the other to the responsible researcher. In case of refusal you will not suffer any penalty.

- This is a research developed under the responsibility of the researcher Alex Lopes dos Reis, who is under the supervision/guidance of the main researcher Raphael Gonçalves de Oliveira, from the State University of Northern Paraná - Health Sciences Center - UENP.

- The objective of this work is to verify the effects of stretching in a physical conditioning program based on Pilates exercises for 8 weeks on flexibility, strength, resistance and muscular power. Pilates exercises are composed of exercises for stretching and strengthening the body's musculature and are usually performed on equipment characteristic of the method, namely: Combo chair, Cadillac trapeze, Reformer, Ladder Barrel. Stretching exercises are usually performed at the beginning of the session, in a dynamic way, that is, repetitions of the same movement are performed up to the limit of the range of motion available, with support from the segment to be worked on the surface of the equipment to maintain balance during execution. Strengthening exercises are performed with the aid of springs that offer resistance to the exercises. Pilates exercises can increase the flexibility and strength of your muscles, improving your quality of life, especially in the face of everyday activities.

- By participating in this work, you will be contributing to identify how the stretching exercises performed initially in Pilates sessions affect people's physical capacities, which will help professionals who work with this type of exercise to plan sessions according to the objective to be prioritized, in addition to being able to obtain the following benefits for your health: a) increase in muscle strength; b) increase in muscle stretching, improving range of motion.

- At the beginning and at the end of the study you will undergo physical tests to assess your flexibility, strength, endurance and muscle power. At the end of each session you will answer questions about your level of satisfaction and perceived effort.

- Interventions with Pilates exercises will take place three times a week, with each session lasting 50 minutes maximum, over 8 weeks. For your safety, all evaluation and intervention procedures adopted in this study will follow international recommendations.

- Two different types of intervention will be carried out, one of which consists of stretching and muscle strengthening exercises and the other only of muscle strengthening exercises. To find out which intervention you will participate in, a raffle will be held.

- The procedures to which you will be submitted will not cause moral, financial or religious damage.

- In order to participate in this study, you must meet the following criteria: a) be between 18 and 45 years old; (b) have a body mass index (BMI) between 18.5 and 24.9 kg/m2; (c) not participating in physical exercise programs for at least six months; (d) being healthy, according to the Physical Activity Readiness Questionnaire (PAR-Q) (THOMAS; SHEPHARD, 1992); (e) not reporting any medical restrictions on physical exercise; (f) have no history of injury, trauma or illness in the last six months; (g) not having undergone previous surgery in the last six months; (h) not having musculoskeletal, cardiorespiratory and neurological disorders that prevent the performance of evaluation and intervention protocols; (i) not being under the action of drugs that

cause muscle relaxation or that may inhibit muscle tonic action; (j) not to use dietary supplements or anabolic steroids; (k) not being on a calorie-restricted diet.

- If you experience any discomfort during the study, such as pain in any part of the body, dizziness or cramps for three consecutive interventions or in one third of interventions within the same month, you must leave the project as a safety measure for your health.

- During evaluations and interventions, there may be risks of pain or discomfort in some region of the body, or another bodily injury. If this happens, the team of professionals will provide you with immediate care, with the application of ice to the injury site, in addition to calling the Mobile Emergency Care Service (SAMU). If you need treatment for an injury as a result of this study, you will be treated until you get better at no cost.

- You will not be remunerated for participating in this study, nor will you have any expenses. If you have any expenses, you will be reimbursed for all costs related to your participation in this research;

- You can stop participating in the study at any time, without any prejudice to you.

- As a result of the study, if you feel aggrieved, you may request compensation as provided by law.

- Your name will not be disclosed anywhere, maintaining your privacy.

- At the end of the study you will have access to the results of all tests (tests to verify flexibility, tests of strength, resistance and muscular power and results of affective valence).

- At the end of the study, you will have the right to participate in the intervention that presents the best results regarding the physical capacities studied.

- If you wish, you will be informed about the general results of this survey.

- Any questions or requests for clarifications, you can contact the supervisor responsible for the project: Prof. doctor Raphael Gonçalves de Oliveira, telephone (43) 9 9641-7628, or (43) 3511-2628, Health Sciences Center, Alameda Padre Magno, 841, Nova Alcântara, Jacarezinho-PR.

- For complaints and/or complaints regarding the ethical aspects of the research, contact the UENP Ethics Committee: CEP/UENP. Address: Rod. BR 369, Km 54, PO Box 261 - Bandeirantes-PR CEP 86360-000. Phone (43) 3542-8056.

“In view of the clarifications provided, I (participant's name)____, born on date ______/_______/_________, I accept and agree to participate in the study 'Effects of stretching in a physical conditioning program based on Pilates exercises on flexibility, strength, endurance and muscle power: a randomized clinical trial', as a participant”.

Volunteer's Full Name	Signature	Date
		/ /
Responsible Researcher's full name	Signature	Date
		/ /
